# Synthesis, DNA Binding and Topoisomerase I Inhibition Activity of Thiazacridine and Imidazacridine Derivatives

**DOI:** 10.3390/molecules181215035

**Published:** 2013-12-06

**Authors:** Elizabeth Almeida Lafayette, Sinara Mônica Vitalino de Almeida, Marina Galdino da Rocha Pitta, Eduardo Isidoro Carneiro Beltrão, Teresinha Gonçalves da Silva, Ricardo Olímpio de Moura, Ivan da Rocha Pitta, Luiz Bezerra de Carvalho, Maria do Carmo Alves de Lima

**Affiliations:** 1 Laboratório de Planejamento e Síntese de Fármacos, Departamento de Antibióticos, Universidade Federal de Pernambuco (UFPE), Recife 50670-901, PE, Brazil; E-Mails: elizabeth.almeidalafayette@gmail.com (E.A.L.); marinapitta@gmail.com (M.G.R.P.); irpitta@gmail.com (I.R.P.); 2 Laboratório de Imunopatologia Keizo Asami (LIKA) and Departamento de Bioquímica, Universidade Federal de Pernambuco (UFPE), Recife 50670-901, PE, Brazil; E-Mails: sinara.monica@gmail.com (S.M.V.A.); ebeltrao@hotmail.com (E.I.C.B.); lbcj@hotlink.com.br (L.B.C.J.); 3 Faculdade de Ciências, Educação e Tecnologia de Garanhuns (FACETEG), Universidade de Pernambuco (UPE), Garanhuns 55290-000, PE, Brazil; 4 Laboratório de Bioensaios para Pesquisa de Fármacos, Departamento de Antibióticos, Universidade Federal de Pernambuco (UFPE), Recife 50670-901, PE, Brazil; E-Mail: teresinha100@gmail.com; 5 Universidade Estadual da Paraíba (UEPB), Campus Campina Grande 58429-500, PB, Brazil; E-Mail: ricardo.olimpiodemoura@gmail.com

**Keywords:** thiazacridine, imidazacridine, DNA binding, topoisomerase I inhibitor

## Abstract

Thiazacridine and imidazacridine derivatives have shown promising results as tumors suppressors in some cancer cell lines. For a better understanding of the mechanism of action of these compounds, binding studies of 5-acridin-9-ylmethylidene-3-amino-2-thioxo-thiazolidin-4-one, 5-acridin-9-ylmethylidene-2-thioxo-thiazolidin-4-one, 5-acridin-9-ylmethylidene-2-thioxo-imidazolidin-4-one and 3-acridin-9-ylmethyl-thiazolidin-2,4-dione with calf thymus DNA (ctDNA) by electronic absorption and fluorescence spectroscopy and circular dichroism spectroscopy were performed. The binding constants ranged from 1.46 × 10^4^ to 6.01 × 10^4^ M^−1^. UV-Vis, fluorescence and circular dichroism measurements indicated that the compounds interact effectively with ctDNA, both by intercalation or external binding. They demonstrated inhibitory activities to human topoisomerase I, except for 5-acridin-9-ylmethylidene-2-thioxo-1,3-thiazolidin-4-one. These results provide insight into the DNA binding mechanism of imidazacridines and thiazacridines.

## 1. Introduction

DNA intercalators are among the most important and promising therapeutic agents to treat many diseases such as cancer. The discovery and development of novel therapeutic intercalator agents for the treatment of malignancy are some of the most important goals in modern medicinal chemistry [1]. Despite of the lack of a detailed mechanistic understanding of the intercalation process at the molecular level [2], it is already known that the binding interaction between external molecules and nucleic acids leads to a significant change in their structures and may have an important influence on their physiological functions [3]. In general, guest molecules may associate to DNA by groove-binding, intercalation or by attractive electrostatic interactions [4].

Acridine derivatives have been used for commercial purposes for more than a century and have recently been investigated as DNA intercalators [5]. These molecules are characterized by the presence of a planar polycyclic system presenting three rings and one or two flexible substituent groups and are well-known probes for nucleic acids as well as being relevant in the field of drug development to establish new chemotherapeutic agents [6]. The biological activity of acridines has been attributed to the planarity of these aromatic structures, which can intercalate within double-stranded DNA, thus interfering with cellular functions [7,8,9].

The cytotoxicity of most acridine-based drugs is based on their ability to suppress topoisomerase activity [8,10]. There are two possibilities for an intercalator to influence the topoisomerase activity and thereby suppress the proliferation of the cell: (a) by intercalation; the binding site of the topoisomerase is occupied and formation of the complex between the enzyme and the DNA is hindered; (b) a ternary complex between DNA, intercalator and topoisomerase may be formed which is significantly more stable than the DNA-topoisomerase complex. The stability of the ternary complex may lead to an enhanced lifetime of the cleaved DNA, *i.e.*, the re-ligation of the strands cannot take place and the strand breaks remain permanent. Thus, the topoisomerase acts as an endogeneous poison and may induce apoptosis [11].

Several classes of topoisomerase inhibitors have been introduced into cancer clinics as potent anticancer drugs, such as anthracyclines (daunorubicin and doxorubicin), demethylepipodophyllotoxin (etoposide), quinolone (voreloxin), iminodazoacridinone derivatives (Symadex™) and ICRF-187 (dexrazoxane) that inhibit topo II by trapping topoisomerase−DNA complexes, either intercalating DNA or by catalytic inhibition [11]. However, the camptothecin derivatives are the only FDA-approved topo I targeted anticancer drugs. Two families of non-camptothecin Top1 inhibitors (indenoisoquinoline and dibenzonaphthyridinone derivatives) are in clinical development [11,12]. It is worthwhile to note that both types of topoisomerase have some degree of redundancy in their functions and inhibition activities. However, the inhibition of only one type of topoisomerase is enough to lead cell death by apoptosis [13].

Amsacrine is a 9-anilinoacridine derivative used to treat a wide variety of cancers, including leukemia and lymphomas [14]. It was one of the first DNA-intercalating agents to be considered as a topoisomerase II inhibitor. The significant clinical use of several of these compounds is limited by problems such as side effects, drug resistance and poor bioavailability, which have encouraged further modifications to these compounds. At present, almost all the reported antitumor agents in the acridine series have been derived from the original lead compounds and they have incorporated changes in the substituents or heterocyclic system modifications [15].

A new class of compounds has been synthesized by our group by coupling acridine and a thiazolidine or imidazolidine nucleus to obtain thiazacridine and imidazacridine derivatives, respectively [5,16]. The biological activities of these compounds examined using diverse techniques and based on various mechanisms of action suggested them as a new class of drugs effective in cancer therapy [17,18]. Beyond these promising results (cytotoxic assays) a better understanding of the mechanism of interaction between these compounds and DNA or key enzymes that can be drug targets, such as topoisomerase, is necessary. Recently, the human topoisomerase I inhibition activity of four thiazacridine derivatives in the presence of supercoiled plasmid DNA for all tested concentrations was described. The results suggested that the coupling of acridine with another nucleus can produce topo I inhibitor acridine derivatives [19]. The present paper describes the synthesis, DNA binding study using ctDNA and human topoisomerase I inhibition activity of some new imidazacridine and thiazacridine derivatives.

## 2. Results and Discussion

### 2.1. Chemistry

**Scheme 1:**
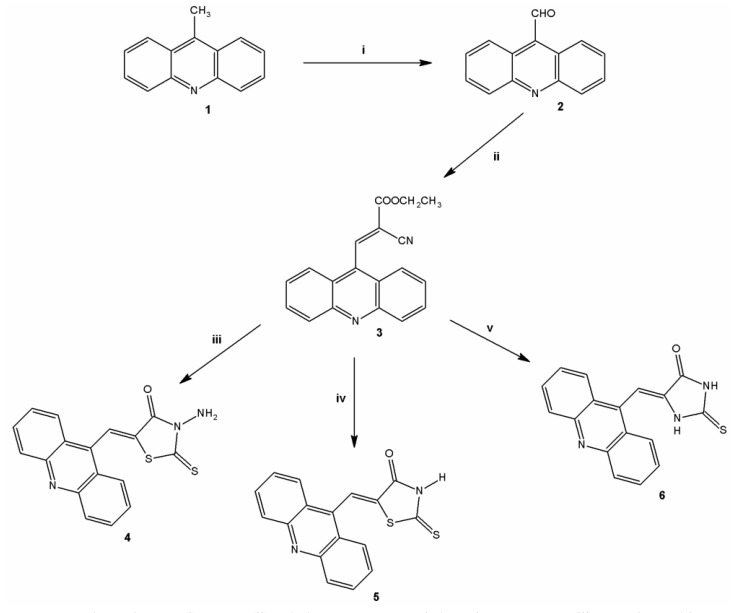
Synthesis of thiazacridine and imidazacridine derivatives.

**Figure 1:**
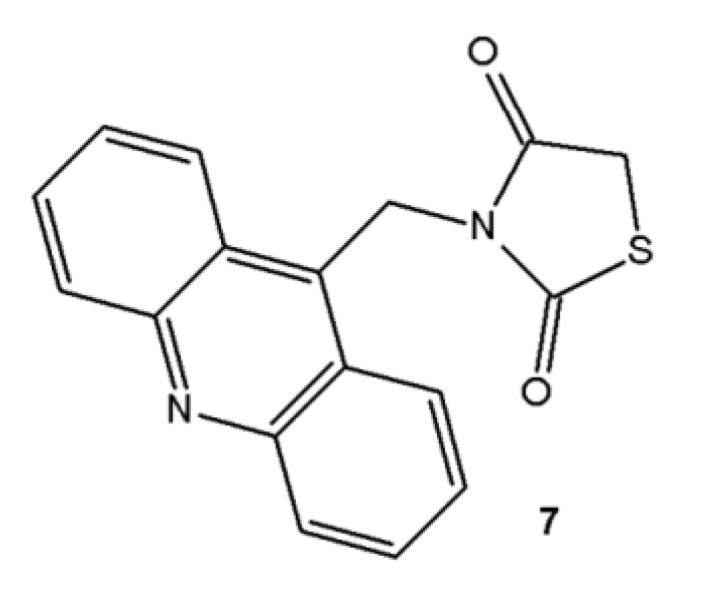
Chemical structure of 3-acridin-9-ylmethylthiazolidine-2,4-dione (**7**).

Derivatives analyzed in this study were all synthesized in the Laboratory of Synthesis and Planning of Drug at the Federal University of the State of Pernambuco (UFPE). The synthesis of the three acridine derivatives **4**‒**6** was performed according to Scheme 1. The compound 9-methylacridine (**1**) was prepared from diphenylamine with zinc dichloride in acetic acid according to Tsuge *et al.* [20]. Subsequently, the oxidation of **1** with pyridinium chlorochromate (PCC) was accomplished according to Mosher and Natale [21] yielding 9-acridinaldehyde (**2**). Next compound **2** was treated with ethyl cyanoacetate to form 2-cyanoacridine-9-yl-acrylate ethyl ester (**3**). In the final step, the intermediate **3** was reacted in absolute ethanol and morpholine with 3-amino-2-thioxo-4-thiazolidinone or 2-thioxo-4-thiazolidinone yielding 5-acridin-9-ylmethylidene-3-amino-2-thioxothiazolidin-4-one (**4**) or 5-acridin-9-ylmethylidene-2-thioxothiazolidi-4-one (**5**), respectively. This same intermediate **3** was reacted with a 2-thioxo-4-imidazolidinone in the presence of ethanol and piperidine to give the derivative 5-acridin-9-ylmethylidene-2-thioxoimidazolidin-4-one (**6**). The purity of these compounds was verified by ^1^H-NMR, ^13^C-NMR, high resolution mass spectrometry and infrared spectroscopy. The synthesis of 3-acridin-9-ylmethylthiazolidine-2,4-dione (**7**) was performed according to Pitta *et al.* [17]. Figure 1 shows the chemical structure of **7**.

*Reagents and conditions*: (i) PCC; (ii) ethyl cyanoacetate, triethyamine, 110 °C; (iii) 3-amino-2-thioxo-4-thiazolidinone, CH_3_CH_2_OH, 50 °C; (iv) 2-thioxo-4-thiazolidinone, CH_3_CH_2_OH, 50 °C; (v) 2-thioxohydantoin, CH_3_CH_2_OH and piperidine, 45–55 °C.

### 2.2. UV-Vis Spectral Absorbance

The interaction of the thiazacridine and imidazacridine derivatives with calf thymus DNA (ctDNA) was monitored by spectrophotometric titrations in Tris-HCl buffer (10 mM, pH 7.6). According to Sabolová *et al.* [22] the UV–Vis spectra of the acridine derivatives showed a significant absorption in the 350–450 nm range, typical for transitions between π-electron energy levels of the acridine ring. Table 1 lists the absorption spectra data of the acridine derivatives in the absence of ctDNA.

In general, upon DNA binding the molecule is positioned in an environment which is different from that of the uncomplexed molecule in solution. Due to these different features the electron distribution is distorted upon π-stacking with the bases. This contributes to the significantly different compound absorption properties in the complexed and uncomplexed forms [4]. Thus, the addition of DNA to a solution of an intercalator results in a characteristic shift of the absorption maximum to longer wavelengths (bathochromic shift or red shift) and a decrease (hypochromicity) or increase (hypercromicity) of the absorbance [23,24,25].

**Table 1:** UV–Vis absorption data of the acridine derivatives.

Compound	λ_max_ free (nm)	λ_max_ bound (nm)	Extinction coefficient (ε) M^−1^	Hypochromicity (%)	Kb M^−1^	Log *p*
4	346	346	10.340	40.43	1.46 × 10^4^	3.37
5	345	345	2.420	0	2.37 × 10^4^	2.93
6	364	368	14.840	28.85	3.25 × 10^4^	3.44
7	361	361	8.000	15.50	6.01 × 10^4^	2.24

**Figure 2:**
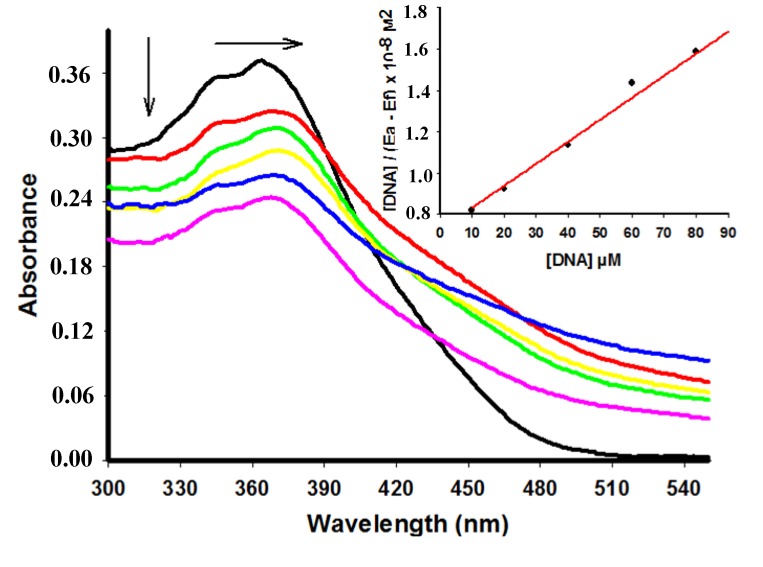
Absorption spectra of derivative **6** (25 µM) with increasing concentrations of ctDNA. [DNA] = 0 (black), 10 (red), 20 (green), 40 (yellow), 60 (blue), 80 (pink) µM. Arrows (**↓**) and (**→**) refer to hypochromic and bathchromic effects, respectively. Inset: corresponding to the plot of [DNA]/(ε_a_ − ε_f_) as function of DNA concentration as determined from the absorption spectral data.

**Figure 3:**
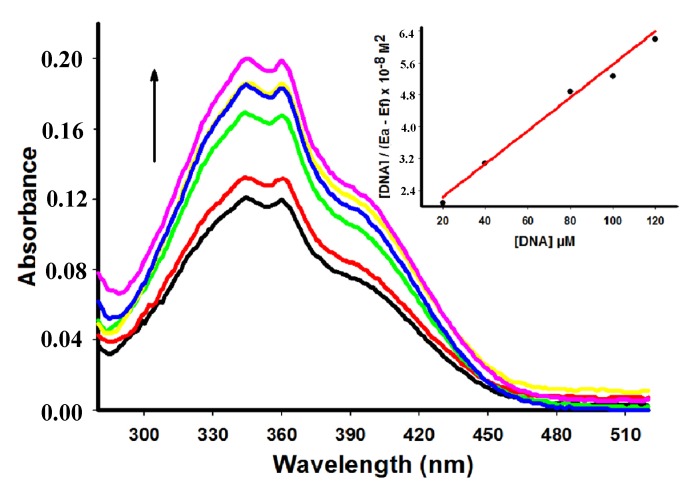
Absorption spectra of derivative **5** (50 µM) with increasing concentrations of ctDNA. [DNA] = 0 (black), 10 (red), 20 (green), 40 (yellow), 60 (blue), 80 (pink) µM. Arrow (**↑**) refers to hyperchromic effect. Inset: corresponding to the plot of [DNA]/(ε_a_ − ε_f_) as function of DNA concentration as determined from the absorption spectral data.

Compounds **4**, **6** and **7** showed a decrease of the peak intensity in the presence of DNA, while DNA did not absorb light in this region. Comparing hypochromism among derivatives compound **4** had the most remarkable decrease of the peak intensity (Table 1). Figure 2 shows the hypochromism effect to the derivative **6** (for the other derivatives see the Supplementary Material). Conversely, addition of increasing amounts of ctDNA to **5** showed a hyperchromism effect (Figure 3) presenting an increase of 52.89% at concentration of 60 µM of ctDNA.

Distinct spectroscopic behaviors stems from the different nature of the substituents located around the main acridine structure that can affect the kinetic and thermodynamic aspects of DNA binding [26]. Absence or presence of an amino moiety at position 3 of the thiazolidin-4-one ring can explain the hypochromic or hyperchromic effect presented by compounds **4** and **5**, respectively. Hyperchromism demonstrated by addition of ctDNA to **5** suggests a strong interaction between the compound and DNA which is different from the classical intercalation binding as demonstrated for metformin (hyperchromism of 9.7%) by Shahabadi and Heidari [25].

Therefore, both hyperchromic and hypochromic effects were spectral features of DNA concerning its double helix structure. The spectral change process reflects the corresponding changes in conformation and structure of DNA after thiazacridine and imidazacridine DNA binding. Hypochromism results from the contraction of DNA in the helix axis as well as from the conformational change of DNA; in contrast, hyperchromism derives from damage of the DNA double-helix structure [27,28].

In addition to the hypochromic phenomenon, a small bathochromic shift of **6** was also observable in the spectra. Figure 2 shows a red shift (Δλ = 4 nm) changing the maximum intensity peak from 364 to 368 nm. Differently, no red shift was observed for the other compounds. Hypochromic and bathochromic effects indicate that compound **6** may bind to DNA and form stable complexes by intercalation mode through the stacking of DNA bases [7].

The absorbance intensity change was used to calculate the DNA binding constants (K_b_) of the acridine derivatives according to McGhee and von Hippel (Table 1) [29]. Typical binding constants for intercalation complexes between organic dyes and DNA range from 1 × 10^4^ to 1 × 10^6^ M^−1^ and are usually significantly smaller than the binding constants of groove binders (1 × 10^5^ to 1 × 10^9^ M^−1^) [4]. The high K_b_ value of **7** suggests a stronger binding towards DNA.

Intercalation has been generally considered as a result of a hydrophobic aromatic molecule (intercalator) displacement from the aqueous hydrophilic to the hydrophobic environment of the DNA pair of bases [30]. Groove binding compounds generally contain unfused aromatic ring systems linked by bonds with torsional freedom. As a consequence the molecules adopt appropriate conformations to fit the helical curvature of the groove without significant perturbation of the DNA [24]. The rotational freedom between the acridine ring and the substituent moiety depends on the spacer features. Compound **7** possesses a methylene bridge linking the acridine ring and the thiazolidine-2,4-dione moiety. Therefore it is supposed that its high flexibility permits the necessary substituent rotation before intercalation or a better conformation for groove-binding. Differently, the other derivatives exhibit a double bond group as spacer that may result a restriction in conformational freedom [31].

Studies of the binding constants of acridine-imidazolidinone derivatives showed that depending on the nature of the alkyl substituents on the imidazolidinone ring, the binding constants decrease with increasing mass of alkyls in the order: ethyl > propyl > butyl > pentyl > hexyl [8]. Amsacrine-DNA link analysis has shown that the binding constant of the formed complex was Kb = 1.2 × 10^4^ M^−1^, indicating weak or moderate strength of binding between them [15].

The cytotoxicity of compounds can be associated with their DNA-binding properties, but also with their hydrophobicity interference as determined by measuring the octanol-water partition coefficient (log *p*) [8]. It was verified that the imidazolidone acridine derivatives with strong cytotoxic effects showed the highest values of log *p*. Herein, a partial investigation of the cytotoxic behavior of the produced acridine derivatives was performed using the log of *p* value (Table 1).

Among the compounds presented here the derivative **7** has already been tested for anticancer activity in a cell toxicity assay against human cancer cell lines [17]. Association between anticancer activity and log *p* value of derivative **7** is consistent with analysis performed by Janovec *et al.* [8].

### 2.3. Fluorescence Emission Spectra

**Figure 4:**
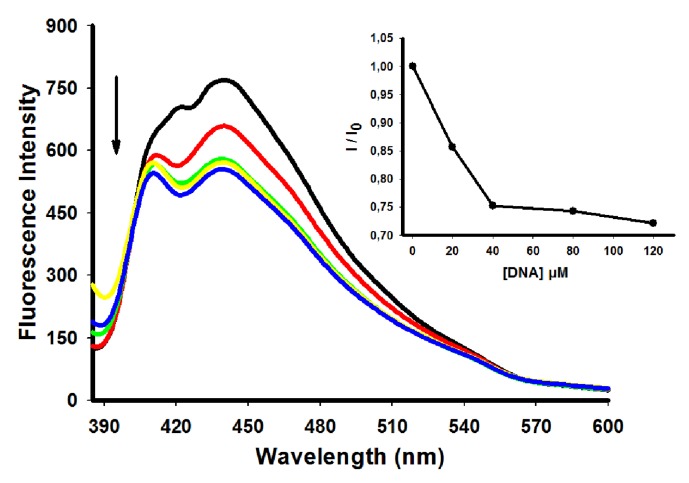
Fluorescence spectra of derivative **5** (10 µM) with increasing concentrations of ctDNA. [DNA] = 0 (black), 20 (red), 40 (green), 80 (yellow) e 120 (blue) µM. Insert: corresponding the fluorescence intensity of bound derivative to ctDNA (I)/fluorescence intensity of free derivative (I_0_).

The binding properties between acridine derivatives and ctDNA were also investigated by fluorescence spectroscopy. These interactions can be monitored either by a “light up” effect (increase on its fluorescence intensity upon binding) or, in most cases, by a “light off” effect (fluorescence decrease after binding). Differences in fluorescence properties of the complexes are influenced by substituents at the peripheral sides of the acridine derivatives [24]. Fluorescence emission was detected after equilibrium reached an optimum level. Upon binding to DNA the fluorescence of all compounds was efficiently quenched by the DNA bases as depicted for the derivative **5** (Figure 4) (other derivatives can be seen in the Supplementary Material).

Table 2 summarizes the fluorescence emission from the derivatives under investigation. The derivative **5** showed the highest decrease in fluorescence emission, while derivative **7** presented the smaller decrease. Emission-quenching phenomena reflect the interaction between the derivatives and ctDNA, consistent with the electronic absorption spectroscopy results [7].

**Table 2:** Fluorescence emission data of acridine derivatives in the presence of ctDNA.

Compoud	λ excitation (nm)	λ emission (nm)	I/I_0_
4	360	415	1.67
5	356	440	2.27
6	364	418	1.11
7	360	435	1.0

Analysis of DNA binding of the compound 9-amino-6-chloro-2-methoxyacridine (ACMA) showed that fluorescence quenching of ACMA by DNA is informative of intercalation, whereas the absorption spectrum may shed light into possible external binding. It was suggested that the DNA-ACMA interaction is not a simple one and that both the intercalative as external binding can be present [26]. In this way, the kind of interaction of the acridine derivatives produced is consistent both with intercalation as external binding.

### 2.4. CD Spectroscopic Analysis

**Figure 5:**
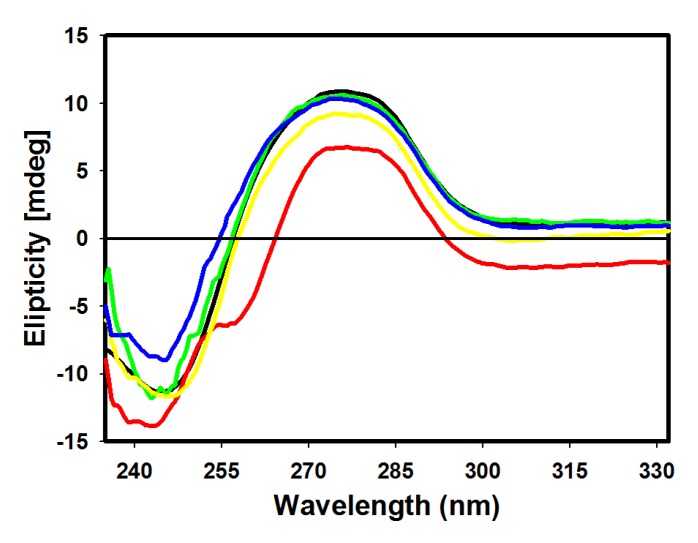
Circular dichroism spectra of ctDNA (100 µM) in 10 mM Tris HCl buffer pH 7.6, in the presence of **4** (red), **5** (green), **6** (yellow) and **7** (blue) derivatives.

Circular dichroism (CD) was used to monitor conformational changes after binding of derivative to ctDNA [10]. CD spectrum of ctDNA shows a positive band (273 nm) due to stacking interactions of DNA bases and a negative band (245 nm) characteristic of ellipticity of DNA [32]. Figure 5 depicts changes in these bands in the presence of acridine derivatives, evidencing derivative-ctDNA complex formation.

Changes of positive bands were observed for all derivatives, with a higher decrease of molar ellipticity for derivatives **5** and **6**. Larger changes were verified at 245 nm, where the most pronounced perturbations were observed in derivatives **4** and **7**. ctDNA binding with derivative **4** produced a decrease of intensity and a small blue shift, while binding with derivative **7** resulted in an increase of molar ellipticity. Mild perturbations in both bands were observed for derivative **5**. Intercalating complexes which disrupt interactions between DNA bases and weaken base stacking should cause a decrease in intensity of CD bands. Reductions in molar ellipticity at a remarkably negative band are associated with destabilization and helix unwinding [33]. Classical intercalation reactions tend to alter the intensity of the two bands due to strong stacking interactions of nucleotide bases and more stable conformations (right-handed B conformations of ctDNA), whereas simple groove binding and electrostatic interactions show a lower perturbation or no perturbation effect whatsoever on the bases’ stacking and ellipticity bands [34].

The CD spectrum of amsacrine (9-anilinoacridine) showed a decrease in intensity at 273 nm indicating an amsacrine aggregation with DNA effect resulting in the DNA helix B conformation disturbance. Such a finding suggests a binding mode which is not a simple intercalative type but a binding where a groove binding is also present [15].

Depending on their structure, compounds can preferentially bind by either groove binding or by intercalation. In the case of unfused-ring systems that do not possess co-planarity within the intercalator-DNA complex inherent the base pair twist may be complemented. It is worth noting that in the vast majority of the unfused compounds studied their intercalation is concomitantly accompanied by groove binding. Conversely, if groove binding is the major interaction mode, experimental evidence has demonstrated that these compounds may also partially interact with base pairs [6]. Therefore, intercalation and groove binding should be viewed as a continuum. The mode with the most favorable free energy for a particular ligand will depend on the DNA sequence and conformation as well as on the specific molecular features of the bound molecules.

### 2.5. DNA Topoisomerase I Inhibition Assay

Topoisomerases are key cellular enzymes that prevent DNA strands from becoming tangled. They cut DNA and cause it to wind and unwind. They play an important role in the transcription, replication and chromosome structure and are regarded as housekeeping genes. Cells die when topoisomerases are inhibited making it targets for the chemotherapy of human cancers [35,36]. Ligands that occupy the topoisomerase binding site may suppress the association of the enzyme with DNA, thus inhibiting its activity [11].

**Figure 6:**
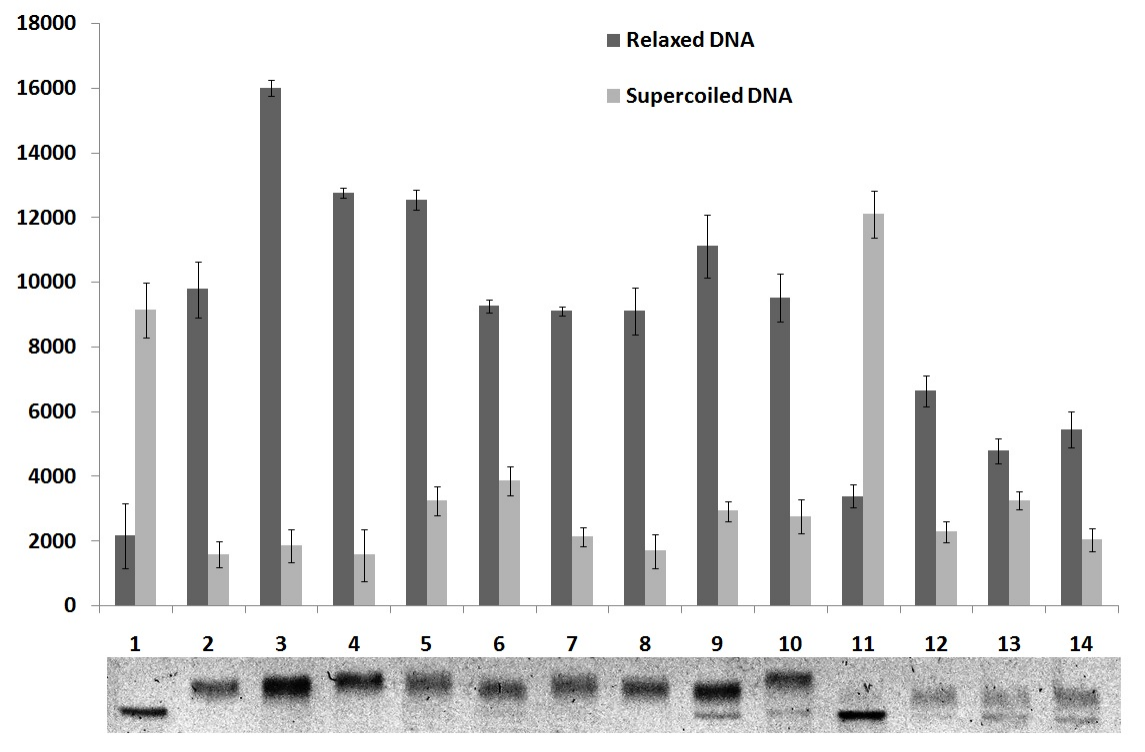
Quantitative analysis of the thiazacridine and imidazacridine derivatives effects on the relaxation of pUC19 DNA plasmid by human topoisomerase 1.

To study the effect of compounds **4**–**7** on the DNA relaxation, supercoiled plasmid pUC19 was incubated with human topoisomerase I in the presence of the compounds in concentrations of 50, 100 and 200 µM. As shown in Figure 6, compounds **6** and **7** showed moderate topoisomerase I inhibitory activity at 100 and 200 μM because super-coiled DNA was partially relaxed by the enzyme. The derivative **4** demonstrated inhibitory activity only at 200 μM. Compound **5** did not show inhibitory activity. These findings suggest that these derivatives have promising structures for the development of topoisomerase I inhibitors and the coupling of acridine with both thiazolidine as imidazolidine nucleus can render potent therapeutics agents as previous demonstrated for thiazacridine derivatives [19]. Some topoisomerase inhibitors such as voreloxin, anthracyclines and iminodazoacridinone derivatives present DNA intercalation power that contributes for their inhibitory activity [11]. Since the derivatives presented here showed inhibitory activity only at high concentration one can be suppose that DNA intercalation is important for this activity.

Lane 1, DNA pUC19 and lane 2, topo 1 + DNA pUC19 (controls). Lanes 3–6, topo 1 + DNA pUC19 + compounds **4**–**7** (50 µM), respectively; lanes 7–10, topo 1 + DNA pUC19 + compounds **4**–**7** (100 µM), respectively, and lanes 11–14, topo 1 + DNA pUC19 + compounds **4**– **7** (200 µM), respectively.

## 3. Experimental

### 3.1. Materials and Instrumentation

Melting points were measured in capillary tubes on a Quimis Model 340.27 apparatus (Quimis, Diadema, Brasil). Thin Layer Cromatography (TLC) was performed on silica gel 60 F254 plates from Merck (Darmstadt, Germany) with fluorescent detection at 254 nm. Infra-red (IR) spectroscopy were recorded on a Bruker IFS66 spectrometer (Bruker, Berlin, Germany), using KBr pellets. The proton (^1^H NMR) and carbon (^13^C-NMR) NMR experiments were performed on Varian Model Plus Spectrophotometer (Varian, Santa Clara, CA, USA) at 300 MHz, in DMSO-*d_6_* as solvent. Mass spectra were recorded on an LCMS-IT-TOF liquid chromatograph-mass spectrometer (Shimadzu, Kyoto, Japan). UV–Vis spectra were measured on an Ultraspec 3000 PRO UV-Visible spectrophotometer (Biochrom Ltd., Cambridge, UK) and excitation and emission spectra on a JASCO FP-6300 (Jasco Corporation, Tokyo, Japan) spectrofluorometer. Circular Dichroism (CD) measurements were performed in a JASCO-815 spectropolarimeter (Jasco Corporation).

### 3.2. Preparation of the Acridine-Thiazolidines Derivatives **4**–**6**

*5-Acridin-9-ylmethylidene-3-amino-2-thioxothiazolidin-4-one* (**4**). A mixture of 3-acridin-9-yl-2-cyanoacrylate ethyl ester (0.5 g, 1.656 mmol), 3-amino-2-thioxo-4-thiazolidinone (0.2453 g), ethanol (25 mL) and morpholine (25 drops) was stirred for 30 min at 50 °C. The product was a yellow solid. Formula: C_17_H_11_N_3_OS_2_; M.W.: 337.4187 g/mol; Yield: 80.55%; Melting point: 214–216 °C; R*_f_*: 0.44 (*n*-hexane/EtOAc 6:4); ^1^H-NMR (DMSO-*d*_6_): δ 8.72 (s, 1H, CH), 6.00 (s, 2H, NH_2_), aromatic hydrogens: 8.25–8.22 (d, 2H, *J* = 8.4 Hz), 8.14–8.11 (d, 2H, *J* = 8.1 Hz); 7.95–7.89 (m, 2H); 7.71–7.66 (m, 2H). ^13^C-NMR: δ 188.6, 162.5, 148.4, 138.2, 131.6, 131.3, 130.2, 129.0, 127.5, 126.1, 122.8. IR (cm^−1^): 3308.5, 1733.2, 1608.9–1409.8, 1247.0, 760.3. MS (*m/z*): 338.04.

*5-Acridin-9-ylmethylidene-2-thioxothiazolidin-4-one* (**5**). 3-Acridin-9-yl-2-cyanoacrylate ethyl ester (0.5 g, 1.656 mmol), 2-thioxo-4-thiazolidinone (0.22053 g), ethanol (25 mL) and morpholine (25 drops) were used for the synthesis. The reaction mixture was stirred for 30 min at 50 °C. The product was a light green solid. Formula: C_17_H_10_N_2_OS_2_; M.W.: 322.4041 g/mol; Yield: 36%; Melting point: 244–246 °C; R*_f_*: 0.66 (*n*-hexane/EtOAc 1:1); ^1^H-NMR (DMSO-*d*_6_): δ 9.35 (s, 1H, NH), 8.48 (s, 1H, CH), aromatic hydrogens 8.24–8.22 (d, 2H, *J* = 8.4 Hz), 8.15–8.12 (d, 2H, *J* = 8.4 Hz), 7.94–7.88 (m, 2H), 7.72–7.66 (m, 2H). ^13^C-NMR: δ 167.28, 167.26, 148.41, 138.92, 137.13, 131.31, 130.21, 127.53, 126.18, 122.76. IR (cm^−1^): 2968.76, 1711.0, 1614.7–1475.9, 1211.4, 758.59. MS (*m/z*): 323.03.

*5-Acridin-9-ylmethylidene-2-thioxoimidazolidin-4-one* (**6**). 3-Acridin-9-yl-2-cyanoacrylate ethyl ester (0.260 g, 0.086 mmol), 2-thioxoimidazolidinone (0.1 g), ethanol (6 mL) and piperidine (8 drops) were used. The reaction mixture was keept at temparature of 45–55°C under stirring. The product was orange solid. Formula: C_17_N_11_N_3_OS; M.W.: 305.3537 g/mol. Yield: 75%. Melting point: 250 °C (carbonized). R*_f_*: 0.66 (*n*-hexane/EtOAc 6:4). ^1^H-NMR (DMSO-*d*_6_): δ 12.43 (s, 1H, NH), 11.65 (s, 1H, NH); aromatic hydrogens: 8.22–8.19 (d, 2H, *J* = 8.7 Hz), 8.10–8.07 (d, 2H, *J* = 8.7 Hz), 7.90–7.85 (m, 2H), 7.67–7.62 (m, 2H), 7.26 (s, 1H, CH). ^13^C-NMR: δ 179.5, 164.3, 148.0, 137.7, 134.3, 130.1, 129.5, 126.2, 125.9, 124.0, 104.3. IR (cm^−1^): 3188.8–3056.1, 2784.4‒2659.8, 1658.5, 1436.7–1342.0, 1193.4, 759.7–656.0. MS (*m/z*): 306.0674.

3.3. UV–Vis Absorption Measurements

UV–vis spectra titrations were performed using 0.01 M Tris buffer, pH 7.6. Calf thymus DNA (ctDNA) was purchased from Sigma (Saint Louis, MO, USA) and used without further purification. The solution of ctDNA in Tris buffer was sonicated for 5 min and the DNA concentration was determined using the molar extinction coefficient 6,600 M^−1^ cm^−1^ at 260 nm [37]. The purity of DNA was determined by monitoring the value of the A_260_/A_280_ ratio. DNA concentration was expressed as micromolar equivalents of the base pairs. Thiazacridine and imidazacridine derivatives were dissolved in methanol or DMSO at a concentration of 1 mM (stock solution) from which working solutions of concentrations ranging from 10 to 50 μM were prepared by dilution using Tris buffer. After compound concentration optimization ctDNA titration was performed with constant acridine derivative concentrations. All measurements were performed at 25 °C in a rectangular quartz cuvette with a 1 cm path length. The intrinsic binding constant (Kb) was obtained by fitting the data to Equation (1) [29]:

[DNA]/(ε_a_ − ε_f_) = [DNA]/(ε_b_ − ε_f_) + 1/K_b_ (ε_b_ − ε_f_) (1)

where ε_a_, ε_b_ and ε_f_ are the apparent, bound, and free extinction coefficients, respectively. Plot fitting of [DNA]/(ε_a_ − ε_f_) *vs.* [DNA] used K_b_ obtained from the ratio of the slope to the Y intercept. The binding data were fitted using the SigmaPlot 10.0 software.

### 3.4. Fluorescence Measurements

Fluorescence measurements of non-bound derivatives were performed with solution concentrations ranging from 10 to 25 μM in 0.01 M Tris buffer, pH 7.6. Emission spectra were recorded in the region 380–700 nm using an excitation wavelength of 356–364 nm. All measurements were performed at 24 °C in a rectangular quartz cuvette with a 1 cm path length. Fluorescence intensities were expressed in arbitrary units. Fluorescence titrations were conducted by the addition of increasing amounts of ctDNA (0–120 μM bp) directly into the cell containing solutions of the derivatives.

### 3.5. Circular Dichroism

Circular Dichroism (CD) measurements were performed (five scans at a speed of 200 nm min^−1^ and collection in 0.1 nm steps) in a 1 cm path-length quartz cuvette at 20 °C. Spectra (from 230 to 300 nm) were determined for derivatives (25 μM) prepared in 10 mM Tris buffer, pH 7.6 in the absence or presence of 100 μM of ctDNA, 10 min after mixing. Buffer was used as blank.

### 3.6. DNA Topoisomerase I Inhibition Assay

Human topoisomerase I inhibition activity was determined using 100 ng of pUC19 DNA plasmid (from Sigma) and 1.0 unit of recombinant human (TOP1 7150) DNA topoisomerase I (Sigma-Aldrich) in relaxation buffer (0.01 M Tris-HCl, pH 7.5, 0.05 M KCl, 5 mM MgCl_2_, 0.1 mM Na_2_EDTA, 0.01% bovine serum albumin) after incubation with or without derivatives for 45 min at 37 °C. Thiazacridine and imidazacridine derivatives tested concentrations were 50, 100 and 200 μM. Agarose gel (0.8%) electrophoresis was performed in SB buffer (NaOH + boric acid) at 7 V/cm for 4 h. Bands were stained with ethidium bromide (1 mg/mL) and photographed under UV light. Quantitative analysis of the gel bands (supercoiled and relaxed DNA) of topoisomerase I inhibition assay was performed by using image processing software (Adobe Photoshop CS4 11.0x2007, San José, CA, USA).

### 3.7. Determination of log p Values

Derivatives’ log *p* values were obtained using the program ChemSketch 12.0 from Advanced Chemistry Development (Toronto, ON, Canada) [38].

## 4. Conclusions

The DNA binding properties of the imidazacridine and thiazacridine derivatives 5-acridin-9-ylmethylidene-3-amino-2-thioxothiazolidin-4-one; 5-acridin-9-ylmethylidene-2-thioxothiazolidin-4-one; 5-acridin-9-ylmethylidene-2-thioxoimidazolidin-4-one and 3-acridin-9-ylmethyl-thiazolidin-2,4-dione were examined by absorption, fluorescence and CD spectra, besides human topoisomerase I inhibition activity. Experimental results indicate that the derivatives can bind to ctDNA both by intercalation or external binding. The products proved intrinsic binding constants with ctDNA ranging between 1.46 × 10^4^ and 6.01 × 10^4^ M^−1^. Fluorescence studies showed emission-quenching phenomenon confirming the interaction between derivatives and ctDNA. CD results presented local perturbations of B conformation of DNA due to derivative binding. In relation to the human topoisomerase I assay, compound **5** had no inhibitory activity, whereas derivative **4** presented inhibitory activity only at 200 μM. Compounds **6** and **7** presented inhibition activity at 100 and 200 μM. These compounds presented the highest intrinsic binding constants with ctDNA with K_b_ values of 3.25 × 10^4^ and 6.01 × 10^4^ for derivatives **6** and **7**, respectivly. These results suggests that topoisomerase I inhibitory activity of these compounds is due to their DNA intercalation power. Finally, these results provided insights into the DNA binding mechanism of imidazacridine and thiazacridine derivatives and their topoisomerase inhibitory ability.

## Supplementary Materials

Supplementary materials can be accessed at: http://www.mdpi.com/1420-3049/18/12/15035/s1.
