# Supplementary material for: Synthesis, DNA Binding and Topoisomerase I Inhibition Activity of Thiazacridine and Imidazacridine Derivatives
Source: Molecules. 2013 Dec 6;18(12):15035–50. doi: 10.3390/molecules181215035 (PMC6270168; doi:10.3390/molecules181215035)

# Supplementary Materials

**Figure S1.** Absorption titration of derivative **4** (50 µM) with increasing concentrations of ctDNA. [DNA] = 0 (black), 10 (red), 20 (green), 40 (yellow), 60 (blue), 80 (pink), 100 (light blue) e 120 (gray) µM. Arrows (↓) refer to hypochromic effects. Inset: corresponding to the plot of [DNA]/($\varepsilon_a$ − $\varepsilon_f$) as function of DNA concentration as determined from the absorption spectral data.

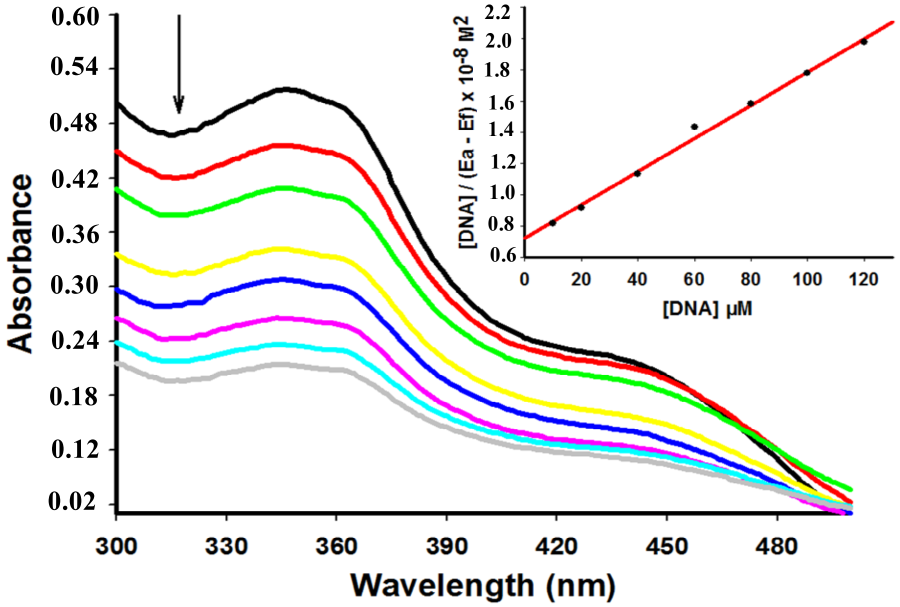


**Figure S2.** Absorption titration of derivative **7** (25 µM) with increasing concentrations of ctDNA. [DNA] = 0 (black), 20 (red), 40 (blue), 60 (yellow), 100 (green) e 120 (pink) µM. Arrows (↓) refer to hypochromic effects. Inset: corresponding to the plot of [DNA]/($\varepsilon_a$ – $\varepsilon_f$) as function of DNA concentration as determined from the absorption spectral data.

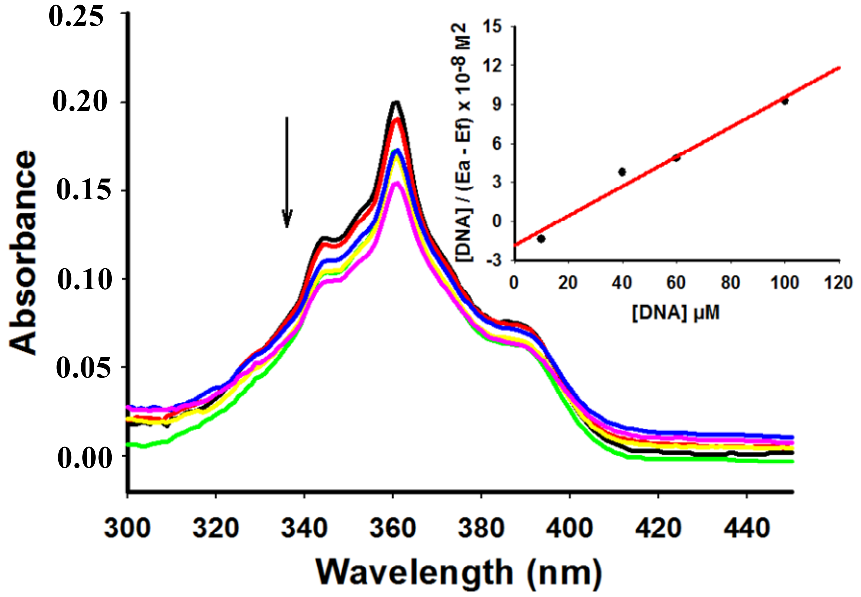

**Figure S3.** Fluorescence changes of derivative **4** (10 µM) with increasing concentrations of ctDNA. [DNA] = 0 (black), 20 (red), 40 (green), 80 (blue) e 120 (pink) µM. Insert: corresponding the fluorescence intensity of bound derivative to ctDNA (I) / fluorescence intensity of free derivative ($I_0$).

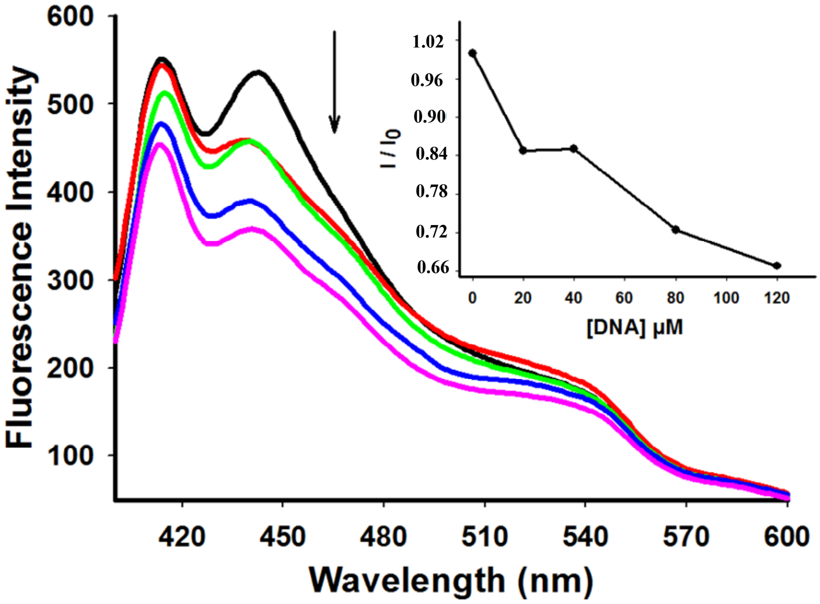


**Figure S4.** Fluorescence spectra of derivative **6** (10 µM) with increasing concentrations of ctDNA. [DNA] = 0 (black), 20 (red), 40 (green), 80 (yellow) e 120 (blue) µM. Insert: corresponding the fluorescence intensity of bound derivative to ctDNA (I) / fluorescence intensity of free derivative ($I_0$).

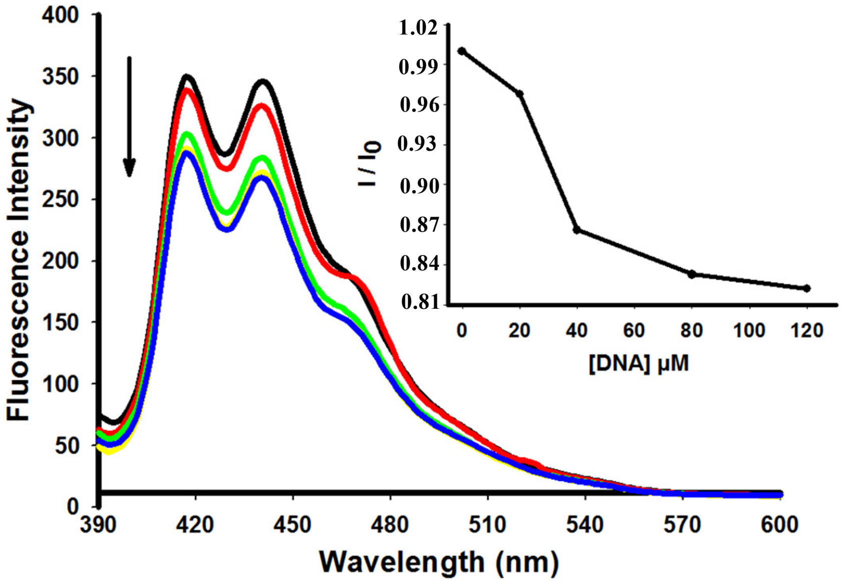

**Figure S5.** Fluorescence changes of derivative **7** (10 μM) with increasing concentrations of ctDNA. [DNA] = 0 (black), 20 (red), 40 (green), 80 (yellow) e 120 (blue) μM. Insert: corresponding the fluorescence intensity of bound derivative to ctDNA (I) / fluorescence intensity of free derivative ($I_0$).

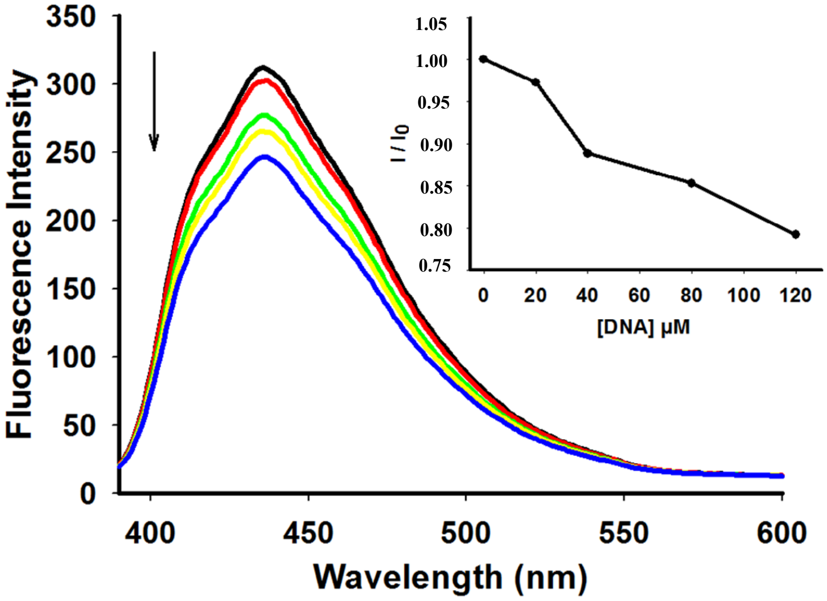


**Figure S6.** Absorption (blue), excitacion (black), emission (red) spectra of derivative **4**, at concentrations 50, 10 and 10 μM, respectively, in Tris-HCl buffer. Excitation at 360 nm and emission at 415 nm.

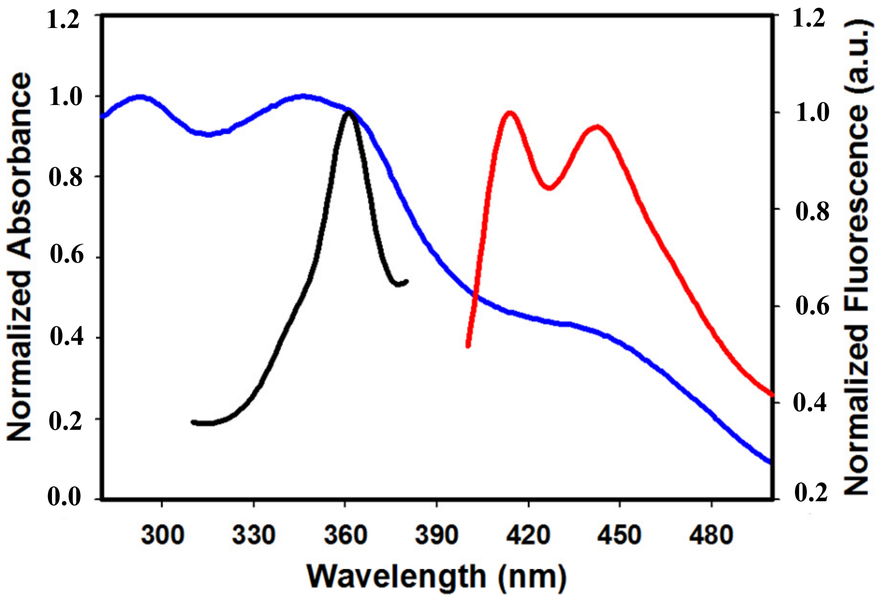

**Figure S7.** Absorption (blue), excitacion (black), emission (red) spectra of derivative **5**, at concentrations 50, 10 and 10 µM, respectively, in Tris-HCl buffer. Excitation at 356 nm and emission at 440 nm.

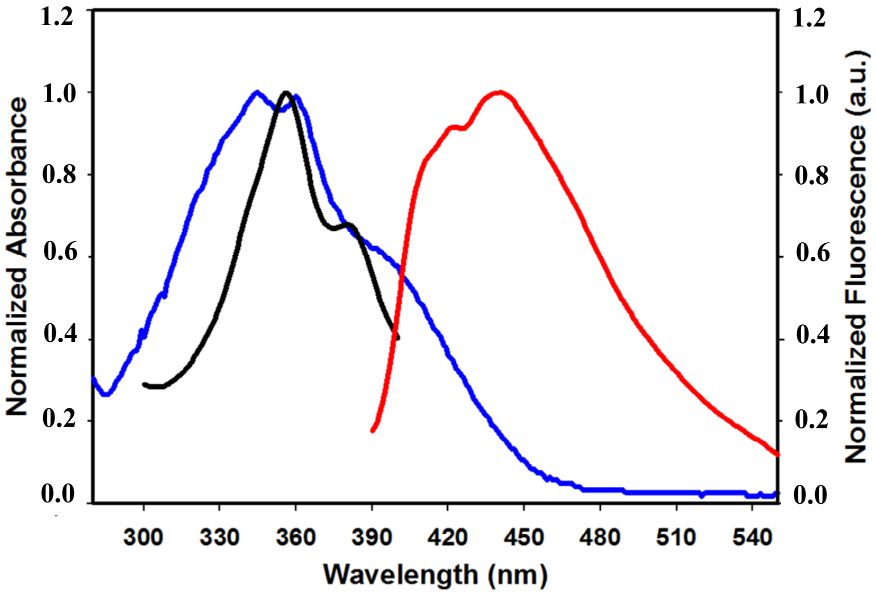


**Figure S8.** Absorption (blue), excitacion (black), emission (red) spectra of derivative **5**, at concentrations 25, 10 and 10 µM, respectively, in Tris-HCl buffer. Excitation at 364 nm and emission at 418 nm.

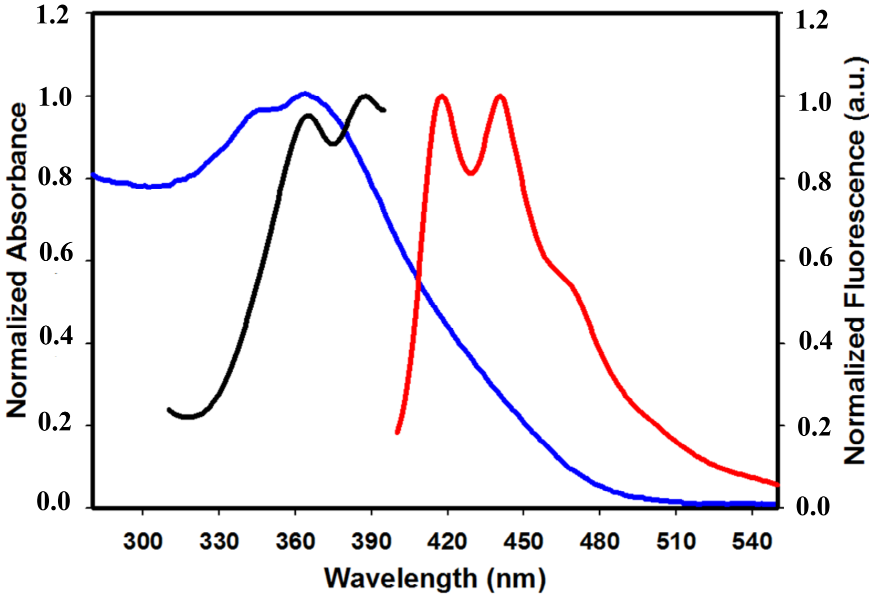

**Figure S9.** Absorption (blue), excitacion (black), emission (red) spectra of derivative **5**, at concentrations 25, 10 and 10 μM, respectively, in Tris-HCl buffer. Excitation at 360 nm and emission at 435 nm.

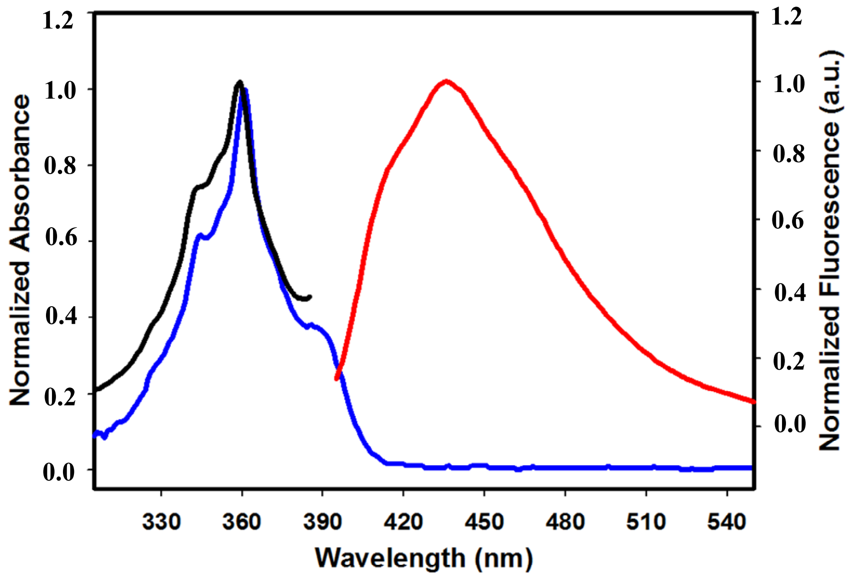


**Figure S10.** $^{13}$C-NMR spectrum (DMSO) of derivative **4**.

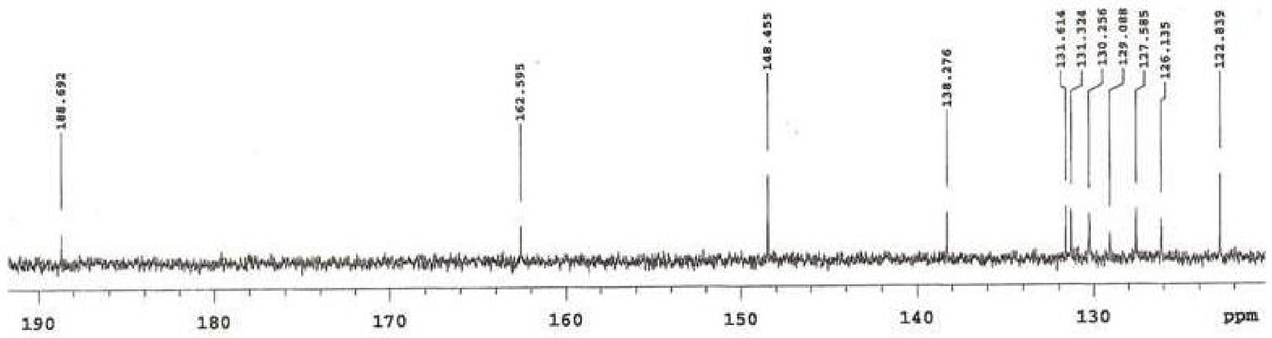


**Figure S11.** $^{1}$H-NMR spectrum (DMSO) of derivative **4**.

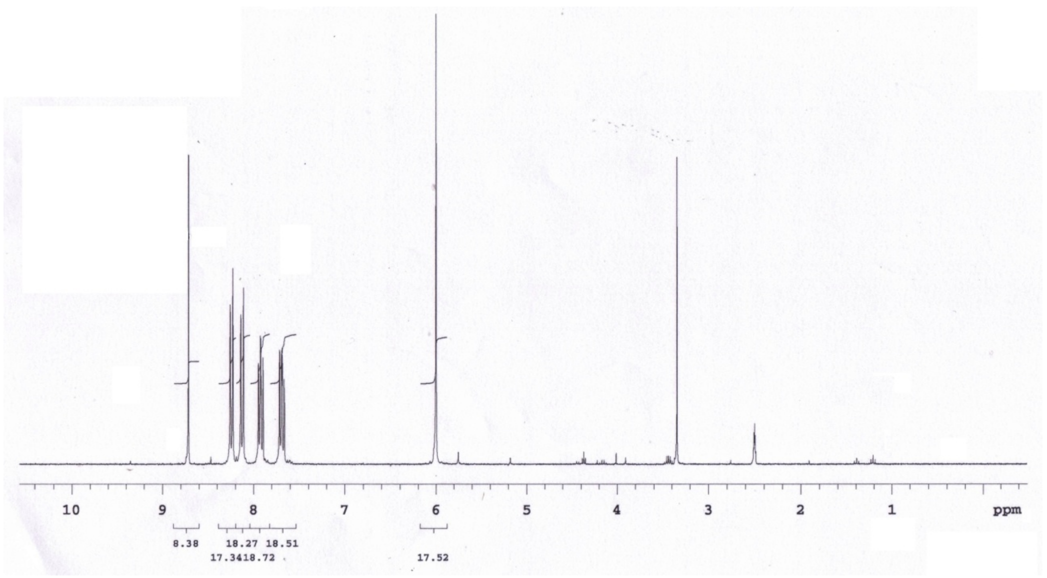

**Figure S12.** $^{13}$C-NMR spectrum (DMSO) of derivative **5**.

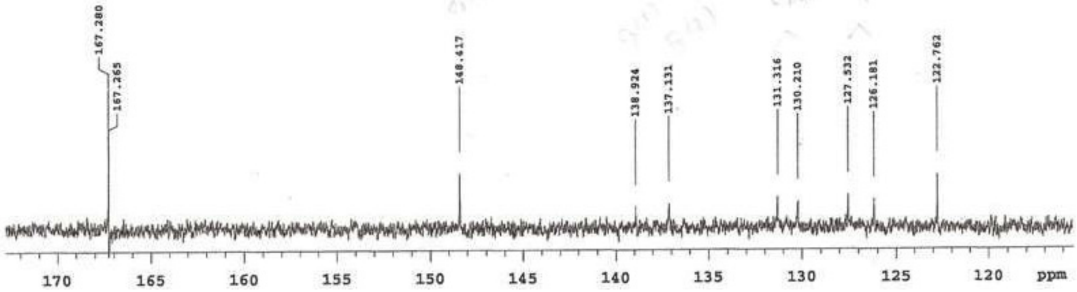


**Figure S13.** $^{1}$H-NMR spectrum (DMSO) of derivative **5**.

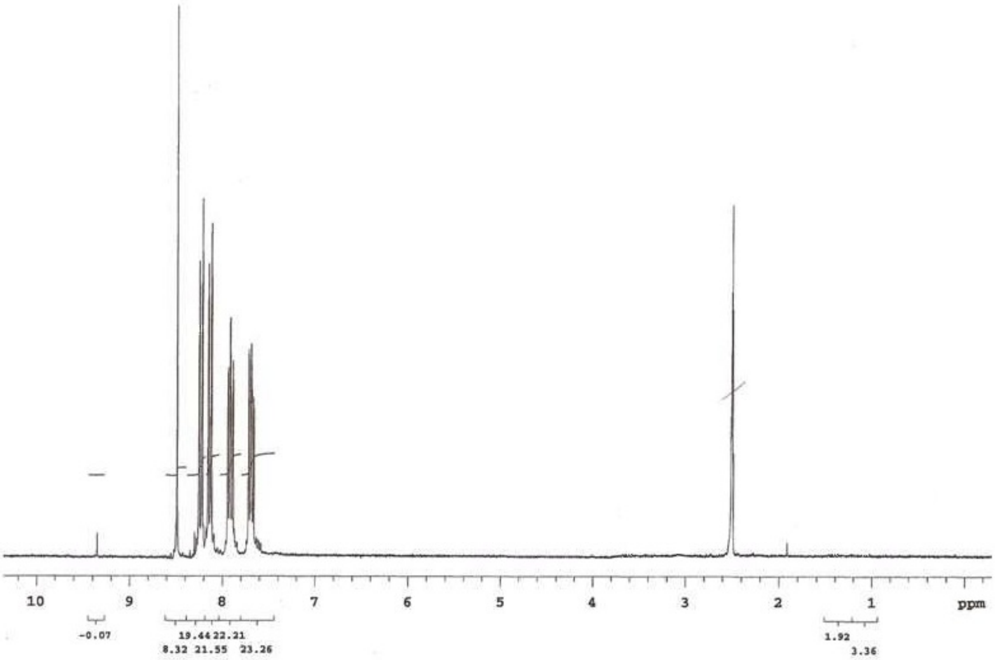


**Figure S14.** $^{13}$C-NMR spectrum (DMSO) of derivative **6**.

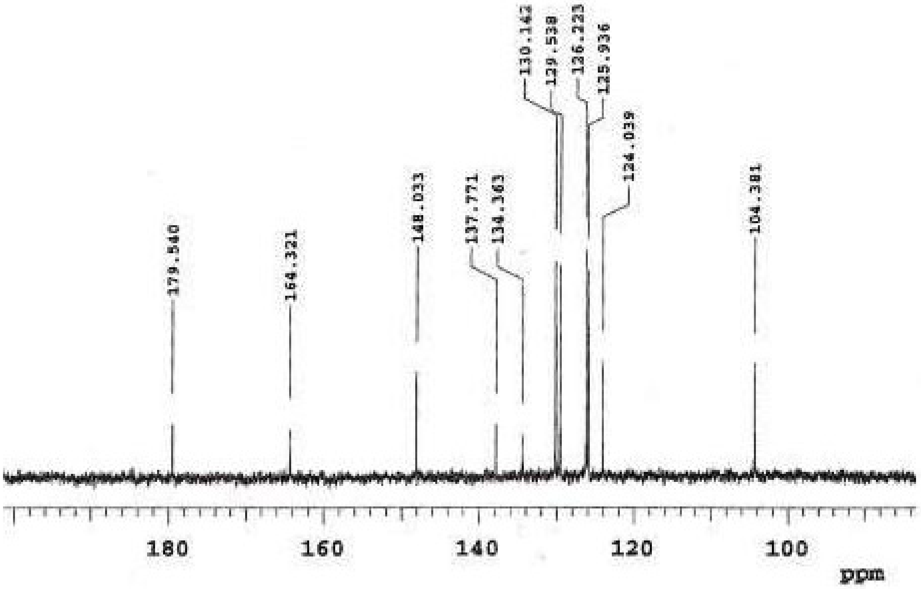

**Figure S15.** $^{1}$H-NMR spectrum (DMSO) of derivative **6**.

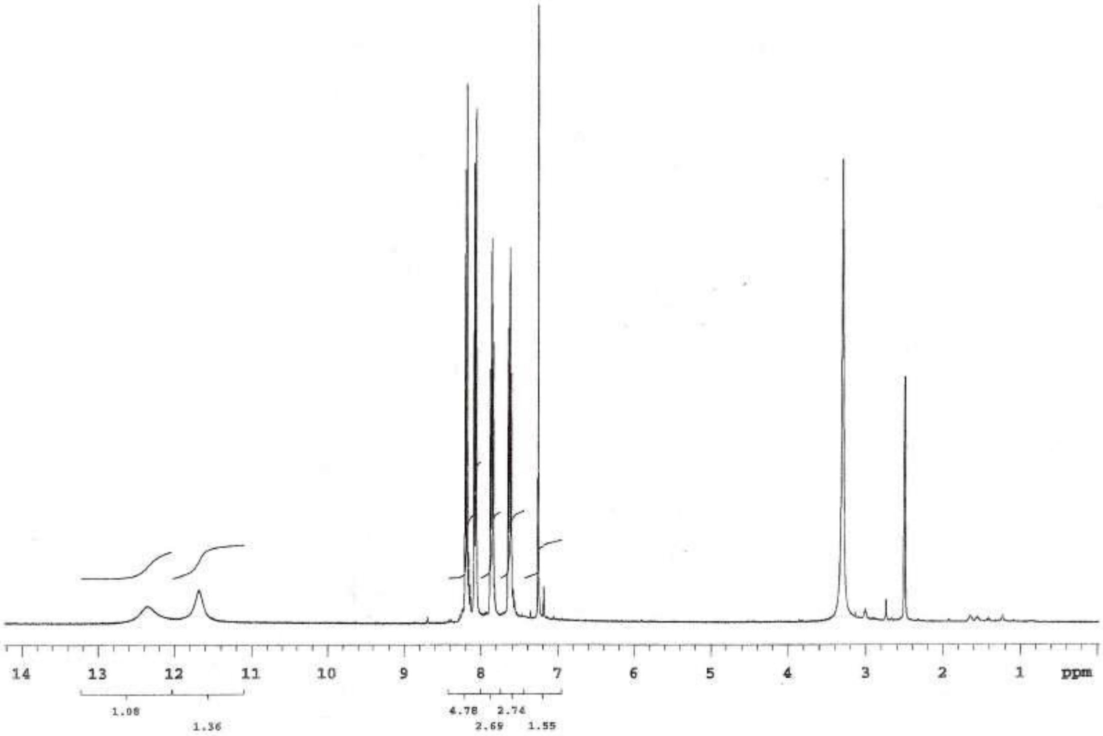

Supplement: Supplementary file 1 [file molecules-18-15035-s001.pdf]
